# Differential response of distinct copepod life history types to spring environmental forcing in Rivers Inlet, British Columbia, Canada

**DOI:** 10.7717/peerj.12238

**Published:** 2021-10-18

**Authors:** Desiree Tommasi, Brian P.V. Hunt, Evgeny A. Pakhomov

**Affiliations:** 1 Institute of Marine Sciences, University of California Santa Cruz, Santa Cruz, CA, USA; 2 Department of Earth, Ocean and Atmospheric Sciences, University of British Columbia, Vancouver, BC, Canada; 3 Institute for the Oceans and Fisheries, University of British Columbia, Vancouver, BC, Canada; 4 Hakai Institute, Heriot Bay, BC, Canada

**Keywords:** Copepod, Life history, Functional traits, Phenology, Demography, Fjord

## Abstract

The temporal dynamics of five copepod species common to coastal waters of the Pacific Northwest were examined in relation to variability in spring temperature and phytoplankton dynamics in 2008, 2009, and 2010 in Rivers Inlet, British Columbia, Canada. The five species were differentiated by life history strategies. *Acartia longiremis*, *Metridia pacifica*, and *Paraeuchaeta elongata* remained active over most of the year. By contrast, the reproductive effort of *Eucalanus bungii* and *Calanus marshallae* was concentrated over the spring period and they spent most of the year in diapause as C5 copepodites. A delay in the timing of the spring bloom was associated with a shift in the phenology of all species. However, following the delay in spring bloom timing, recruitment to the G_1_ cohort was reduced only for *E. bungii* and *C. marshallae*. Recruitment successes of *E. bungii* and *C. marshallae* was also drastically reduced in 2010, an El Niño year, when spring temperatures were highest. Reasons for the observed differential response to spring environmental forcing, and its effect on upper trophic levels, are discussed.

## Introduction

Organisms have evolved diverse life history strategies to maximize their fitness in different environments (Hairston & Bohonak, 1998). Generally, resources in any environment are limited and organisms have to make a choice in allocation resources to growth, maintenance, and reproduction to maximize their fitness (Hairston & Bohonak, 1998). Differences in such life history decisions are particularly evident in the overwintering adaptations of temperate and polar marine zooplankton. Temperate and polar systems are characterized by strongly seasonal physical and biological cycles. Most of the yearly phytoplankton production is focused in the spring or fall blooms (Longhurst, 1995), leading to a short feeding season and long periods of limited resources. Zooplankton have evolved different strategies to contend with the long intervals of diminished food availability in these environments. Atkinson (1998) describes two life history strategies adopted by different copepods to survive the winter period and exploit the short, but productive feeding season. One group, Type I, diapauses in winter as late stage copepodites and focuses reproduction in the spring period (Atkinson, 1998). They possess large lipids stores that are used for metabolic maintenance during diapause, maturation to the adult stage, and, for some species, initiation of egg production prior to the onset of the spring phytoplankton bloom. Thus, for Type I copepods, capital breeding, which is reproduction focused on stored resources (Varpe et al., 2009), may contribute to the yearly total reproductive effort. Type II copepods have a longer reproductive season, a broader diet, and less reliance on diapause to overwinter (Atkinson, 1998). These copepods are more likely to rely solely on income breeding, with most of their reproductive effort dependent on concurrent feeding (Varpe et al., 2009). Given the distinctive life history strategies of these two copepod groups, they may respond differently to changes in the seasonal timing of the spring bloom.

In high latitude systems, the onset of the productive spring period occurs when an increase in stratification, due to the seasonal warming of surface waters and a decrease in wind speeds, shallows the mixed layer depth, allowing for an increase in phytoplankton growth rates (Sverdrup, 1953). The timing of such physical spring forcing events varies interannually and is shifting following climate change (Edwards & Richardson, 2004; Mackas, Batten & Trudel, 2007; Bograd et al., 2009; Foreman, Pal & Merryfield, 2011; Mackas et al., 2012). Following such changes in forcing variables, the timing of the onset of the productive spring period is also expected to vary under climate change (Ji et al., 2010). For instance, on the British Columbia shelf, a later start of coastal upwelling has been correlated with a delayed onset of the spring phytoplankton bloom (Borstad et al., 2011). In the Bering Sea, absence of ice in years of higher spring temperatures is associated with a late phytoplankton bloom (Stabeno et al., 2001). It remains to be assessed if a specific type of zooplankton overwintering strategy will be more successful under the observed shifts in the spring timing of physical forcing events and phytoplankton phenology. Will Type I and Type II copepods experience different phenological changes and will one type be more resilient to forecasted future changes in the spring environment?

The mechanisms relating phenological changes in zooplankton to variation in spring environmental conditions are not completely understood and may be region specific. Globally, the seasonal timing of many zooplankton taxa has been observed to occur earlier when spring temperature is warmer (Mackas et al., 2012). Zooplankton phenological data from the offshore Northeast Pacific, focused solely on the Type I copepod *Neocalanus plumchrus*, also point to an earlier biomass peak in association with warmer spring SST (Mackas, Batten & Trudel, 2007; Mackas et al., 2012). However, on the Northeast Pacific shelf, the response of zooplankton to climate change may be more complex than the “earlier when warmer” global trend. In the Bering Sea shelf ecosystem, recruitment of *Calanus marshallae*, the dominant copepod in the area, is highest in years of an early spring bloom (Baier & Napp, 2003). Over the British Columbia shelf, variation in the recruitment of upper trophic levels, believed to be driven by changes in zooplankton phenology, appear to be more strongly related to the onset of upwelling winds and phytoplankton spring bloom timing than spring SST (Borstad et al., 2011). However, zooplankton phenological studies have yet to be conducted in the region to verify which signal, warmer spring temperatures or a later onset of the feeding season following a delayed arrival of upwelling winds, is the most important determinant of zooplankton phenological changes.

Here we examine the seasonal cycle of five copepods species in the coastal environment of the Northeast Pacific: *Acartia longiremis*, *C*. *marshallae*, *Eucalanus bungii*, *Metridia pacifica*, and *Paraeuchaeta elongata*. These five species constitute the majority of copepod biomass in the study area, Rivers Inlet, British Columbia (Tommasi et al., 2013) and possess different life history strategies. Amongst them, *C. marshallae* and *E. bungii* display a Type I overwintering strategy (Osgood & Frost, 1994; Shoden, Ikeda & Yamaguchi, 2005). *Calanus marshallae* descends to overwintering depths in early summer and diapause mainly as C5 copepodites (Harrison et al., 1983; Smith & Vidal, 1986; Osgood & Frost, 1994). Exit of diapause and moulting from C5 copepodites to adults occurs in early winter, prior to the spring bloom (Smith & Vidal, 1986; Osgood & Frost, 1994; Peterson, 1998; Baier & Napp, 2003). Spawning starts before the spring bloom (Smith & Vidal, 1986; Osgood & Frost, 1994; Baier & Napp, 2003) likely using lipid reserves (Osgood & Frost, 1994), and continues over the spring period, peaking at high food concentrations (Osgood & Frost, 1994; Peterson, 1998). In the case of *E*. *bungii*, the majority overwinter as C5 copepodites, but a minority do so as C3 or C4 (Krause & Lewis, 1979; Shoden, Ikeda & Yamaguchi, 2005; Yamaguchi et al., 2010). Molting to the adult stage starts prior to the bloom, in February. However, unlike *C*. *marshallae*, final gonad maturation and spawning are associated with the onset of the spring bloom (Shoden, Ikeda & Yamaguchi, 2005; Yamaguchi et al., 2010; Takahashi & Ide, 2011). Thus, *E*. *bungii* may rely more on income breeding than *C*. *marshallae*.

By contrast, *A. longiremis*, *M. pacifica*, and *P. elongata* display a Type II strategy. The majority of the *M*. *pacifica* population overwinters as adult females, which remain active and feed omnivorously (El-Sabaawi et al., 2009). All copepodite stages appear year-round and reproduction is thought to occur throughout the year (Padmavati, Ikeda & Yamaguchi, 2004; Osgood & Frost, 1994; Batchelder, 1985). *Paraeuchaeta elongata* reproduces year-round and remains active during the winter (Evans, 1973; Ikeda & Hirakawa, 1996). Similarly, *A*. *longiremis* has a long reproductive season, from March to September (Norrbin, 1994). However, only adult females overwinter, and they do so in very low numbers (Norrbin, 1994; Norrbin, 2001).

Specific aims for this study were (1) to present the seasonal cycle of these species in the region; (2) to assess if the phenology of these copepods varied following interannual differences in spring bloom timing and spring temperature; (3) to determine how the interaction between overwintering strategy and spring bloom timing affects recruitment success. We focused our analysis on the spring season as juveniles of upper trophic levels that depend on zooplankton for successful recruitment appear during this period (Borstad et al., 2011).

## Materials and Methods

**Figure 1:**
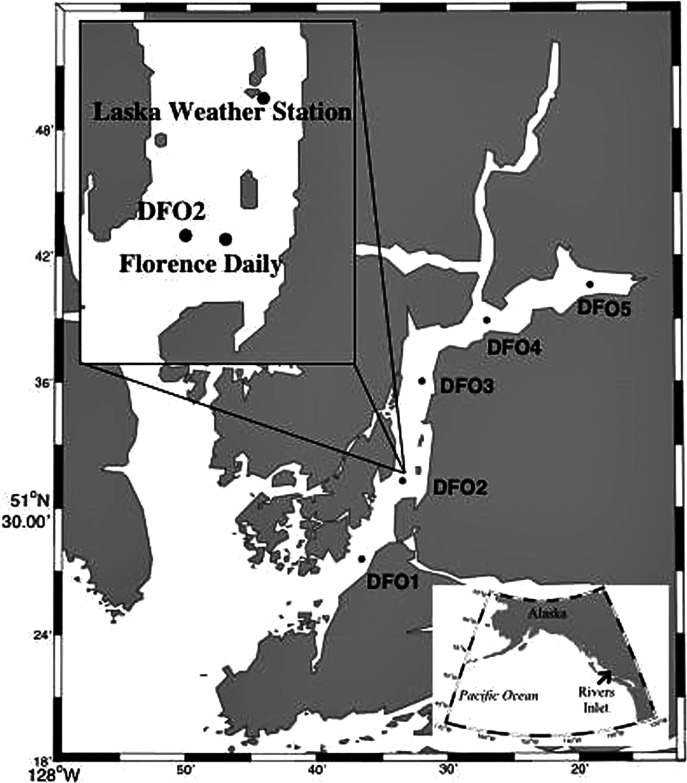
Map of Rivers Inlet showing sampling stations. The inset highlights the position of the Florence Daily sampling station where daily hydrographic data was collected.

Detailed sampling design description can be found in Tommasi et al. (2013, 2014). In short, zooplankton hauls from ~5 m above the bottom (to a maximum depth of 300 m) to the surface were conducted during the day fortnightly (2008 and 2009) or monthly (2010) from March to June with a 150 µm mesh bongo net from the *M. Western Bounty* across five sampling stations equally spaced along the length of Rivers Inlet (51° 26′, 127° 38′), a deep (365 m maximum depth), 45 km long, on average 3 km wide fjord of glacial origin with a 137 m deep sill at its mouth on the Central Coast of British Columbia, Canada (Fig. 1; Table S1). Prior to every bongo net deployment, profiles of temperature, salinity (not plotted), and fluorescence (chlorophyll *a*) were collected for the entire water column with a SBE 25 CTD. Profiles of temperature, salinity, and fluorescence were also taken every morning to a depth of 30 m from the Florence Daily site (Fig. 1). The fluorescence readings (mg m^−3^, factory calibrated) were converted into chlorophyll *a* concentrations using filtered samples (see regressions in Tommasi et al., 2013). No zooplankton samples were collected at DFO2 in the first 2008 cruise and at DFO5 in the late April 2008 cruise. Moreover, during the early June 2009 cruise, only a sample at DFO2 was collected from the *M. Western Bounty*. Samples from the remaining stations were collected from the *M. CCGS J. P. Tully* using a 236 µm bongo net during a routine Cooperative Plankton Research Monitoring Program (COPRA) cruise of the Department of Fisheries and Oceans Canada.

While the majority of the data analysis conducted in this paper is drawn from this March–June dataset, additional full water column zooplankton samples were collected monthly at the DFO2 station (identified as a core station representing entire Rivers Inlet pelagic systems, see Tommasi et al. (2013, 2014)) from July to September in 2008, February and July–August 2009, July 2010, October 2010 to February 2011. Further samples were also collected from the DFO2 station in February 2008, September 2009, and September 2010 from the *M. CCGS J. P. Tully* using a 236 µm bongo net during routine COPRA cruises of the Department of Fisheries and Oceans Canada (Table S1). From these samples, we present the relative, stage specific abundance of the species under study to assess the duration of their reproductive period. Finally, to assess the vertical distribution of the species of interest, additional zooplankton vertical hauls were carried out on every cruise, both during the day and night, in 2010 at the DFO2 station in the following depth intervals: 300-0, 100-0, 30-0, and 10-0 m. The sampling depths were chosen base on vertical profiles of environmental parameters (temperature, salinity, chlorophyll-a). A 2-0 m oblique tow was also obtained to capture copepod naupliar stages due to their concentration in poorly sampled near surface layers.

### Zooplankton taxonomic analysis

Detailed zooplankton taxonomic analysis design description can be found in Tommasi et al. (2013, 2014). In short, copepod adults and copepodites from stages C4 and C5 were identified to species. Copepodites C1 to C3 were identified to family for Calanidae copepods and to species for *M. pacifica*, *E. bungii*, *P. elongata*, and *A. longiremis*. Because *C. marshallae* was the numerically dominant Calanidae copepod in this system (Tommasi et al., 2013, 2014), we assume that most of the Calanidae copepodites identified belong to this species and we refer to them as *C. marshallae* C1–C3 throughout this paper. All references to C6 individuals of all species denote counts of adult females only. Males were identified but not used in the analysis. Zooplankton density was expressed on a unit volume basis (ind. m^−3^). Moreover, zooplankton abundance on a unit area basis (ind. m^−2^) was computed by multiplying the zooplankton density (ind. m^−3^) by the depth of the water column sampled.

*Acartia longiremis* copepodite stages C3 and below, *M. pacifica* copepodites stage C2 and below, and *C. marshallae* stage C1 copepodites are undersampled by the 150 μm mesh net (Nichols & Thompson, 1991). However, as the same undersampling bias was present across years we believe that comparison of seasonal and interannual dynamics are warranted even if absolute values of abundance of these stages need to be treated with care. As compared to the 150 um mesh net, the 236 μm mesh net would also severely undersample *A. longiremis* copepodite stages C4–C5, *M. pacifica* stage C3 copepodites, *C. marshallae* stage C2 copepodites, and *P. elongata* and *E. bungii* stage C1 copepodites (Nichols & Thompson, 1991). Thus, comparisons of the abundance of the aforementioned stages between cruises in 2009 and between years during the early June cruise could not be carried out.

### Determination of development times

Birth dates of the C1 spring cohort and adult (*A*. *longiremis* and *M*. *pacifica*) or C5 cohort (*C*. *marshallae* and *E*. *bungii*) were back calculated using development times. No clear cohort could be distinguished for *P*. *elongata* and no birth date estimates were computed for this species. Copepod development time is temperature dependent and is generally modeled as a Belehrádek-type temperature dependent function (Corkett, McLaren & Sevigny, 1986):

$$D=a(T+\alpha)^\beta$$

where *a* and *α* are species and stage specific parameters. Corkett, McLaren & Sevigny (1986) showed that *β* remained constant, at −2.05, for 11 copepod species. Thus, in our development rate parameterization we assumed a constant *β* of −2.05 for all species and stages.

The *a* and *α* parameters were estimated by fitting the Belehrádek equation to experimentally derived development times obtained from the literature using maximum likelihood in R. Table 1 lists the studies employed and the fitted parameters. Development times were derived from stage C1 onwards. For *E. bungii*, the only experimental investigation of development time was conducted at a single temperature (5 °C, Takahashi & Ide, 2011). Therefore, using a Q_10_ of 2.2 for development rate (development rate = reciprocal of development time) (Hirst & Bunker, 2003), we computed development time at 10 °C before fitting the Belehrádek curve. For *C. marshallae* we used *a* and *α* parameters computed by Campbell et al. (2001) for *C. finmarchicus*, as was done by Ji, Stegert & Davi (2013) and Baier & Napp (2003) in their estimates of *C. marshallae* development. *Calanus finmarchicus* has a similar length, 2.4–4.2 mm, as *C. marshallae*, 2.9–4.5 mm (Frost, 1974).

**Table 1:** Species and stage specific Belehrádek parameters used in the calculation of development times.

Species	Stage	*a*	*α*	Data Source
*Metridia pacifica*	C1	26,829	−18.23	Padmavati & Ikeda (2002)
	C2	37,075	−18.89	
	C3	42,436	−18.21	
	C4	52,728	−18.17	
	C5	55,469	−16.04	
	C6	87,794	−17.08	
*Acartia longiremis*	C1	9,024	−11.16	Landry (1975), Leandro et al. (2006) Data for similar species *Acartia clausii*
	C2	10,419	−11.21	
	C3	10,962	−10.00	
	C4	12,299	−9.86	
	C5	13,128	−9.14	
	C6	15,557	−9.28	
*Calanus marshallae*	C1	5,267	−9.11	Campbell et al. (2001) Data for *Calanus finmachicus*
	C2	6,233	−9.11	
	C3	7,370	−9.11	
	C4	8,798	−9.11	
	C5	10,964	−9.11	
	C6	15,047	−9.11	
*Eucalanus bungii*	C1	34,211	−19.16	Takahashi & Ide (2011)
	C2	44,529	−18.31	
	C3	52,452	−18.66	
	C4	60,421	−18.93	
	C5	62,852	−18.18	
	C6	103,740	−18.90	
*Paraeuchaeta elongata*	C1	80,790	−27.38	Ikeda & Hirakawa (1996), Evans (1973)
	C2	142,073	−30.44	
	C3	123,335	−23.04	
	C4	192,090	−26.71	
	C5	191,994	−23.68	
	C6	713,934	−40.37	

Vertical frequency distribution data in the day and night for zooplankton was available for 2010 at DFO2 in the following layers: 0–2, 0–10, 10–30, 30–100, and 100–300 m. Mean temperature in each zooplankton sampling layer for each cruise/year/site/time of day combination was computed. These temperature data were then input into the Belehrádek function with the fitted stage and species specific *a* and *α* parameters to determine development times in Rivers Inlet for each depth layer/cruise/year/site/species/stage combination. The development time for the entire water column was then computed as an average weighted by the relative abundance of each stage and species in each layer. It was assumed that the zooplankton maintained the same vertical distribution between sites and years. For each cruise/year/site combination the weighted average by length of daylight of the day and night development times estimates was computed. Length of daylight was obtained from tables of standard times of solar rise/set for Port Hardy, British Columbia (127° 25′ W 50° 42′ N) downloaded from the National Research Council of Canada sunrise/sunset calculator (http://www.nrc-cnrc.gc.ca/eng/services/sunrise/advanced.html, accessed January 2013). The estimated species and stage specific development times were comparable to development times reported in the literature (Table 1).

### Determination of egg production rates

To assess if the copepod species under study behaved more like income or capital breeders we compared seasonality in egg production rate (EPR) and numbers of egg produced to the back-calculated birthdate of the G_1_ cohort. We searched the published literature for equations relating egg production rate (EPR) (eggs female^−1^ day^−1^) to chlorophyll concentration (mg m^−3^) (Table 2). The *Eucalanus bungii* relationship between EPR and chlorophyll was derived from a logarithmic regression of the EPR and chlorophyll concentrations reported in Smith & Lane (1991) for *E. californicus* (Appendix 1). For *Metridia pacifica*, Hopcroft et al. (2005) reported a relationship between specific egg production (SEP) (d^−1^) rather than EPR and chlorophyll. To estimate EPR from SEP, SEP estimates were multiplied by the ratio of the dry weight (DW) of Rivers Inlet *M. pacifica* adult females, 136 μg, and the *M*. *pacifica* egg DW, 0.62 μg. Egg DW was estimated from egg carbon weight derived from the egg diameter, 145 μm, (Hopcroft et al., 2005), and egg density, 0.14 × 10^−6^ μg C μm^−3^ (Kiorboe & Sabatini, 1994). Egg carbon weight was then converted to ash free dry weight (AFDW) assuming a 0.4 ratio of carbon to AFDW (Hopcroft et al., 2005) and finally to DW assuming a 0.9 ratio of AFDW to DW (Hirst & Bunker, 2003). Egg production rates estimates were corrected to *in situ* temperature using a Q_10_ of 2.7 (Bunker & Hirst, 2004).

**Table 2:** Egg production rate equations derived from the literature.

Species	EPR equation	Source
*Acartia longiremis*	EPR = 54.01*(1−e^(−0.29*(Chl + 0.17)^)	Dam, Peterson & Bellantoni, 1994 (for *Acartia tonsa*)
*Calanus marshallae*	EPR = 2.7021*Chl + 1.3116 If EPR >50, EPR = 50	Gómez-Gutiérrez & Peterson (1999)
*Eucalanus bungii*	EPR = 19.874*ln(Chl) + 65.414	Smith & Lane (1991) (for *Eucalanus californicus*)
*Metridia pacifica*	EPR = 0.18*Chl^1.95^/(1.02^1.95^ + Chl^1.95^)	Hopcroft et al. (2005)

The equations in Table 2 were used to determine EPR for each cruise/year/site/species combination from *in situ* chlorophyll concentrations as follows. Chlorophyll concentration depth profiles for each cruise/year/site/time of day combination were averaged over each of the vertical stratified zooplankton sampling layers and species specific EPR were obtained for each depth layer/cruise/year/site/time of day combination. The EPR of each layer was then multiplied by the abundance of adult females in each layer to estimates the number of egg produced. These estimates were summed to compute the total number of eggs produced over the entire water column. For each cruise/year/site combination the weighted average by length of daylight of the number of eggs computed during the day and night was computed. Length of daylight was obtained from tables of standard times of solar rise/set for Port Hardy. British Colombia (127° 25′ W 50° 42′ N) downloaded from the National Research Council of Canada sunrise/sunset calculator (http://www.nrc-cnrc.gc.ca/eng/services/sunrise/advanced.html, accessed January 2013).

**Figure 2:**
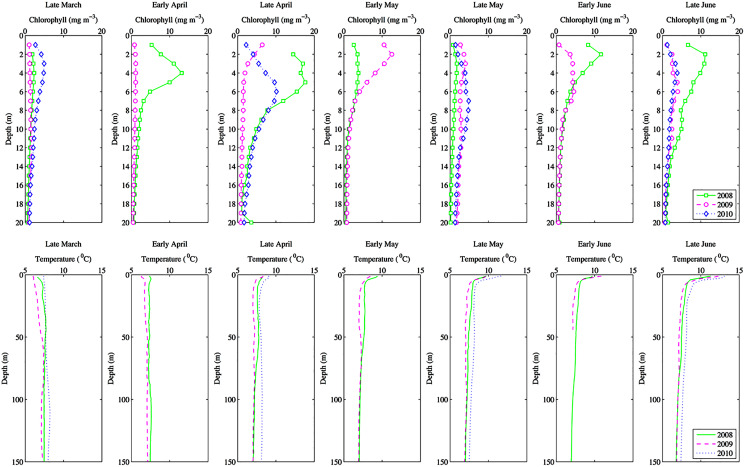
Seasonal depth profiles of chlorophyll (mg m^−3^) and temperature (°C) averaged across inlet stations. Seasonal depth profiles of chlorophyll (mg m^−3^) and temperature (°C) averaged across inlet stations for the 3 years of observations. Note that in 2010 depth profiles were taken monthly rather than fortnightly.

Phytoplankton growth rates are fast and chlorophyll can fluctuate widely over the course of 2 weeks. Therefore, to better assess the seasonal fluctuations in EPR we also determined EPR from the daily chlorophyll time series. Daily chlorophyll depth profiles were only taken down to 30 m, thus EPR for the daily station is an estimate for the first 30 m of the water column. However, as chlorophyll below 30 m is very low (Fig. 2), EPR down to 30 m was a good approximation of total EPR (Appendix 2).

*Paraeuchaeta elongata* is a carnivorous copepod and as such it was felt that chlorophyll would not be an adequate proxy of food consumption and no EPR was computed for this species.

## Results

### Oceanography

**Figure 3:**
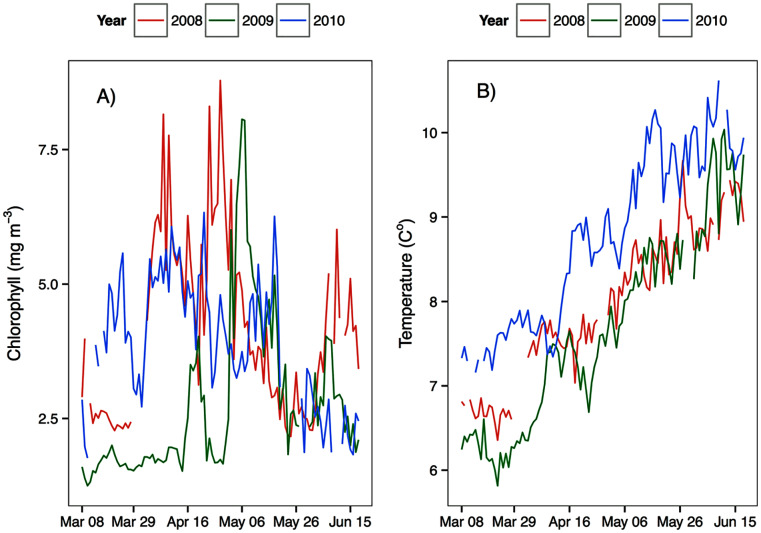
Average upper 30 m chlorophyll (A) and temperature (B) from March to June at the Florence Daily station for the 3 years of observations.

**Figure 4:**
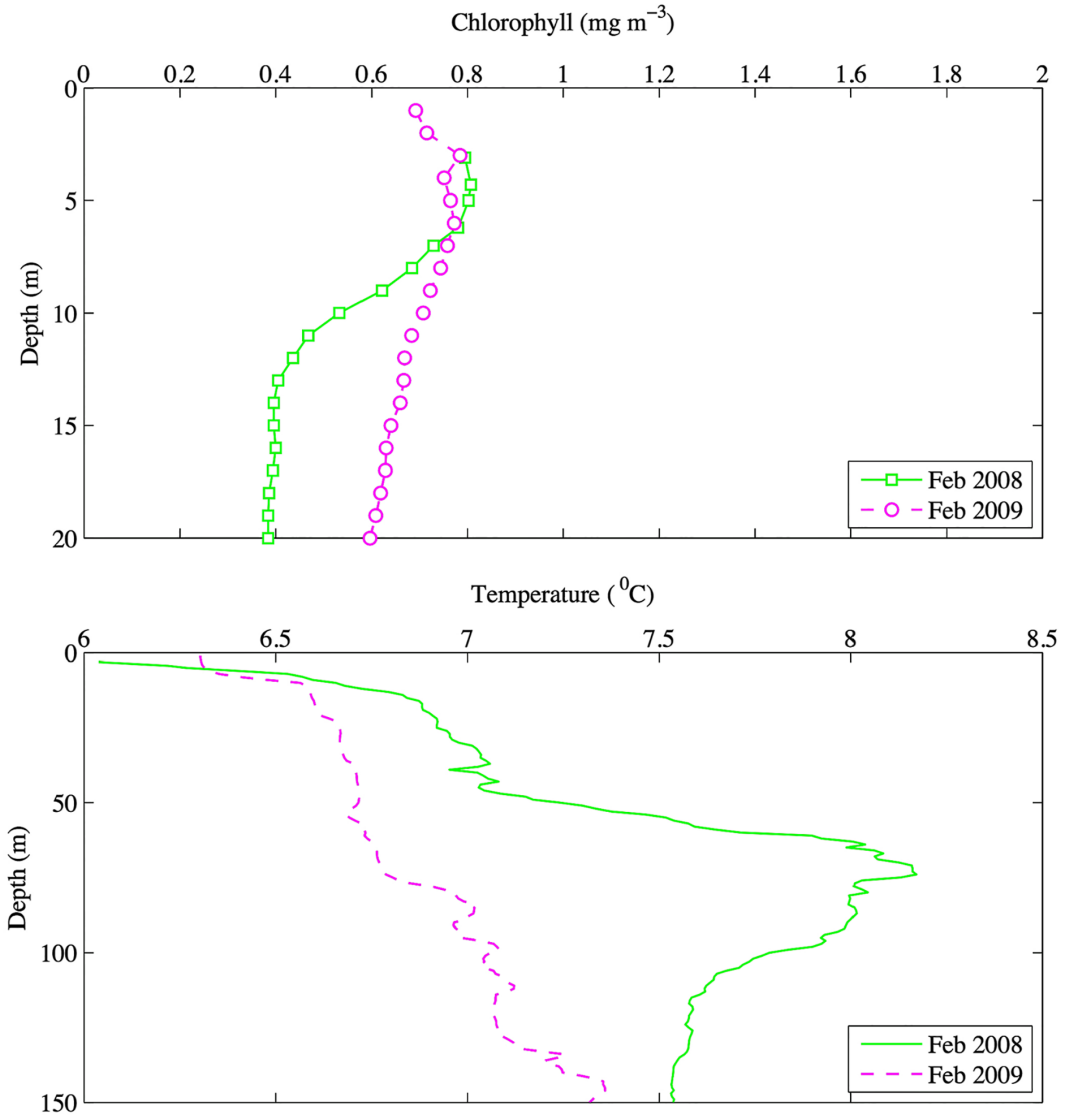
February depth profiles of chlorophyll (mg m^−3^) and temperature (°C) at DFO2 for 2008 and 2009.

Environmental parameters had been presented in detail elsewhere (Tommasi et al., 2013, 2014). Briefly, the spring bloom occurred from early April to early May in both 2010 and 2008 (Figs. 2 and 3). In 2009 it started later, in early May, and was less prolonged (Figs. 2 and 3), even if in February chlorophyll concentration in the top 10 m at DFO2 was comparable between 2008 and 2009 (Fig. 4).

Temperature in 2010, an El Niño year, was consistently higher than both 2008 and 2009 (Figs. 2 and 3). Daily mean 0–30 m temperature did not show a large variation between 2008 and 2009 over most of the sampling season (Fig. 3). Nevertheless, upper 50 m temperatures in March–June 2008 were warmer than 2009 (Fig. 2). DFO2 February temperature profiles revealed that the temperature difference between 2008 and 2009 observed in March was already present in February, and was particularly significant in the intermediate water column (Fig. 4).

### Modeled development times

**Figure 5:**
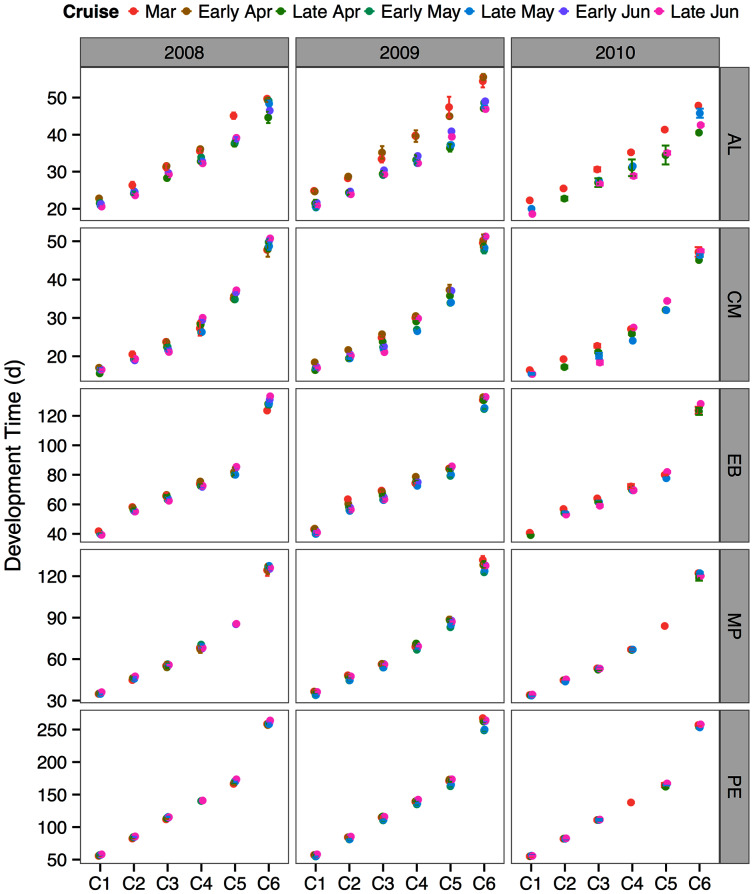
Species and stage-specific estimates of development times averaged across inlet stations computed for each sampling cruise and sampling year. Error bars represent standard errors. AL, *Acartia longiremis*; CM, *Calanus marshallae*; EB, *Eucalanus bungii*; MP, *Metridia pacifica*; PE, *Paraeuchaeta elongata*.

The between year mean temperature variation did not result in large differences in development times between years or across the sampling season (Fig. 5). One exception was the species *A. longiremis*, which showed slightly longer development times in March of all years and in early April 2009 as compared to the rest of the sampling season (Fig. 5).

### Phenology and population structure

**Figure 6:**
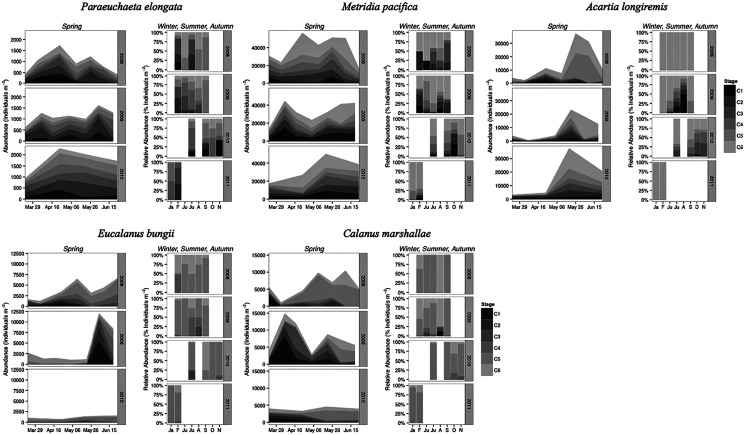
Stage and species-specific variation in abundance averaged across inlet stations over the spring period (March to June), and in relative abundance at DFO2 over the winter, summer, and autumn.

*Paraeuchaeta elongata*: There was no distinct seasonal or interannual trend in the abundance of adult female *P. elongata*, with no clearly discernible cohort of adult females (Fig. 6, upper left panels). However, abundance of C1 varied seasonally and a shift between years in the timing of C1 copepodites peak abundance was observed (Fig. 6). Highest densities of C1 copepodites occurred in late April in 2008 and 2010, but in early May and June in 2009 (Fig. 6). Given a 2 months’ development time to C1 (Table 3), birth dates of this C1 cohort were back calculated to late February in 2008 and 2010 and to early March in 2009.

**Table 3:** Species and stage specific development times and stage durations. Development times represent the days taken to develop to the specified stage and were computed from the species and stage-specific Belehrádek parameters listed in Table 1. Stage durations were computed from the difference of two consecutive development times.

	Development time ± SE (days)						Adult total length (mm)
	C1	C2	C3	C4	C5	C6	
*Acartia longiremis*	22 ± 0.4	25 ± 0.5	28 ± 0.6	34 ± 0.6	40 ± 1.0	48 ± 0.9	0.98–1.25
*Calanus marshallae*	17 ± 0.2	20 ± 0.3	22 ± 0.4	28 ± 0.4	35 ± 0.4	49 ± 0.4	2.9–4.5
*Eucalanus bungii*	41 ± 0.4	57 ± 0.6	64 ± 0.6	73 ± 0.6	82 ± 0.6	129 ± 0.9	6.6–8.0
*Metridia pacifica*	35 ± 0.2	46 ± 0.3	55 ± 0.3	67 ± 0.4	86 ± 0.7	125 ± 0.8	2.5–2.9
*Paraeuchaeta elongata*	57 ± 0.2	84 ± 0.4	114 ± 0.2	139 ± 0.7	169 ± 0.9	259 ± 1.2	6.3–6.5
	**Stage duration ± SE (days)**						
	**C1**	**C2**	**C3**	**C4**	**C5**		
*Acartia longiremis*	3 ± 0.9	3 ± 1.1	6 ± 1.2	6 ± 1.6	8 ±1 0.9		
*Calanus marshallae*	3 ± 0.5	2 ± 0.7	6 ± 0.8	7 ± 0.8	14 ± 0.8		
*Eucalanus bungii*	16 ± 1.0	7 ± 1.2	9 ± 1.2	9 ± 1.2	47 ± 1.5		
*Metridia pacifica*	11 ± 0.5	9 ± 0.6	12 ± 0.7	19 ± 1.1	39 ± 1.5		
*Paraeuchaeta elongata*	27 ± 0.6	30 ± 0.6	25 ± 0.9	30 ± 1.6	90 ± 2.1		

Stage C1 copepodites were always present over the spring-early summer season, implying that reproduction occurred continuously, as is typical of a Type II overwintering strategy copepod (Fig. 6). Abundance data from DFO2 shows that young copepodites (stage C1–C4) were present in the summer as well as in February of all years (Fig. 6). Furthermore, winter data from 2010–2011 demonstrates that young copepodites also occurred in late November (Fig. 6). Thus, reproduction likely occurred throughout the year.

**Figure 7:**
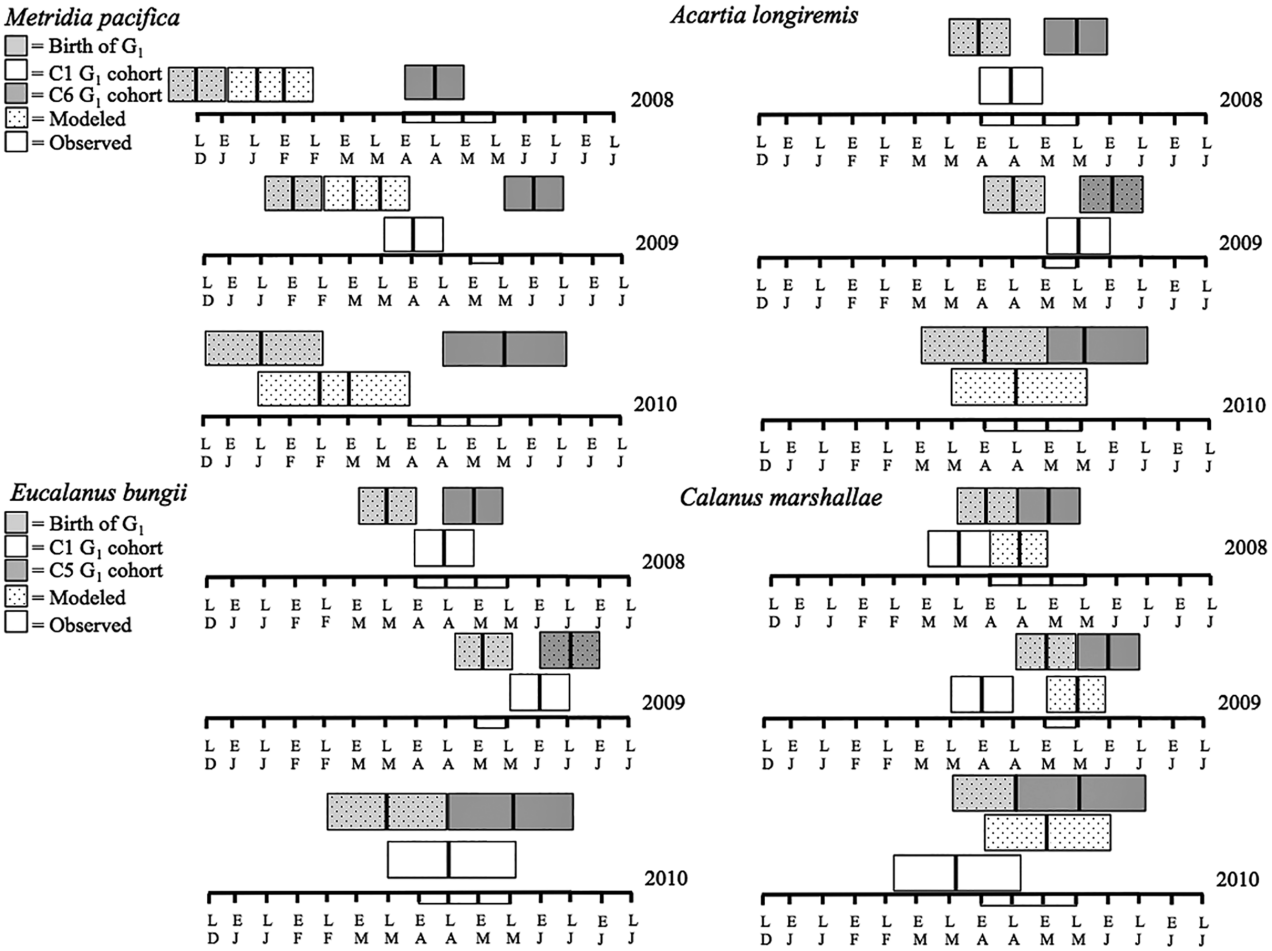
Late December (LD) to late June (LJ) cohort development. Solid boxes represent actual observations. Dotted boxes are back-calculated estimates using development times. In each box, the central black line represents the actual timing of sampling. Boxes were extended 2 weeks (2008 and 2009) or a month (2010) from this date to represent the uncertainty in the cohort appearance resulting from our fortnightly (2008 and 2009) or monthly (2010) sampling resolution. For *M. pacifica*, the C1 cohort has two potential timings because of the almost 2-week duration of the C1 stage. See text for more details. The rectangle on the time axis represents the timing and duration of the spring bloom. Note that if the C1 G_1_ cohort (first cohort of adult females) was not observed within the sampling season timeframe no observed C1 G_1_ cohort is shown.

*Metridia pacifica*: The timing of the appearance of the first cohort of *M. pacifica* adult females (G_1_) shifted between years. G_1_ abundance peaked in late April in 2008, but in early June in 2009 (Fig. 6, upper middle panels). However, the phenology change was not associated with differences in the size of the cohort, which remained comparable between 2008 and 2009 (Fig. 6). Assuming a 4 months’ development time to the adult stage (Table 3), the G_1_ cohort birthdate was in late December in 2008 and in early February in 2009 (Fig. 7, upper left panels). In 2010, adult female abundance was highest in late May, albeit abundances started increasing in late April (Fig. 6). Numbers of adult females in 2010 were less than in 2008 and 2009 (Fig. 6). However, total population size was highest in 2010 and lowest in 2008 (Fig. 6). Peak female abundances in 2008 and 2009 were short lived (less than 2 weeks) (Fig. 6), thus the monthly sampling frequency of 2010 may have missed peak 2010 female abundances.

The seasonal timing of the first C1 copepodite cohort also differed between years. In 2009, densities of stage C1 copepodites fist increased in early April (Fig. 6). The development time from stage C1 to C6 is calculated as a range, with the maximum as the difference in development time between stage C1 and C6 and the minimum as the difference in development time between stage C2 and C6. For *M*. *pacifica*, this results in a range of 79–90 days (Table 3). Thus, considering our 2-week sampling frequency, the early April 2009 stage C1 abundance peak (Fig. 6), likely resulted in the June 2009 cohort of G_1_ females (Fig. 7). Since adult females’ numbers peaked in late April 2008 and in late May in 2010 (Fig. 6), the first peak in stage C1 copepodites in 2008 likely occurred in late January-early February, and in 2010 in early to late March (Fig. 7). No between year variability in the appearance of the second cohort of stage C1 copepodites was observed. It occurred in late May in all years (Fig. 6).

As copepods with a Type II overwintering strategy, *M. pacifica* appeared to have a long reproductive season, lasting from January to October. Data from DFO2 showed that stage C1 and C2 copepodites were already present in February of all years (Fig. 6), implying that reproduction had already started in late December or January. Reproductive activity lasted throughout the summer, with juvenile stages (C1–C3) present throughout the spring-early summer across inlet stations (Fig. 6), and at DFO2 until September (Fig. 6). Winter sampling from 2010 indicated that stage C1 copepodites were present until late October (Fig. 6).

*Eucalanus bungii*: The phenology of *E. bungii* shifted between years. Peak abundance of C1 and C2 copepodites stages occurred in late April in both 2008 and 2010 but in early June in 2009 (Fig. 6, lower left panels). Similarly, the G_1_ stage C5 cohort abundance appeared in early May 2008 and late May 2010, but only in late June 2009 (Fig. 6). This timing is consistent with a development time of 41 days from stage C1 to C5 (Table 3). The back calculated birth date of the G_1_ C1 cohort was late March for 2008 and 2010, and early May for 2009 (Fig. 7, lower left panels). Abundance of the C5 cohort was higher in 2008 as compared to 2009 and 2010 (Fig. 6), implying that recruitment success to the diapausing cohort may have been more successful in 2008.

*E. bungii* behaved as a Type I overwintering strategy copepod, showing a short reproductive season and diapausing over the winter months. Its reproductive activity, when *E. bungii* non-diapausing stage C1 and C2 were present, was focused between March–May in 2008 and 2010, and in June in 2009 (Fig. 6). By July of all years the population was dominated by C5 copepodites, presumably in diapause (Fig. 6). A second reproductive event appears to have occurred in the late summer of each year. We observed an increase in the relative abundance of adult females as compared to stage C5 copepodites in late July 2008 and 2009, and in September 2010 (Fig. 6). This spawning activity may have resulted in the August and September 2009 increase in C3 and C4 copepodites abundance and in the November 2010 rise in numbers of C4 copepodites (Fig. 6). Winter data from 2010–2011 showed that the population diapaused mainly as stage C5 copepodites from October–January (Fig. 6). In February of all years some stage C5 copepodites had already started molting into adults (Fig. 6). The fraction of adults in the population in February 2008 was higher than in 2009 (Fig. 6), but by late March of both years most of the C5 had molted into adults (Fig. 6).

*Calanus marshallae*: *Calanus marshallae* displayed a distinct change in phenology between years. Maximum abundances of stage C5 copepodites occurred in early May 2008 and late May 2010, but in early June in 2009 (Fig. 6, lower right panels). Assuming 18 days development time from stage C1 to C5 (Table 3), the early June 2009 C5 G_1_ cohort would have developed from stage C1 copepodites present in late May, while the early May 2008 stage C5 G_1_ copepodites would have developed from late April stage C1 copepodites, and the 2010 May G_1_ stage C5 cohort would have resulted from late April stage C1 copepodites (Fig. 7, lower right panels). Even considering the fortnightly sampling frequency in 2008 and 2009, there is a discrepancy, especially marked in 2009, between the appearance of maximum C1 copepodites abundance and the back-calculated timing of appearance of the G_1_ C1 cohort (Fig. 7). It appears that early season copepodite mortality is high, and that only the C1 cohort whose appearance was timed with the spring bloom recruited successfully to stage C5. As for *E. bungii*, abundance of the C5 cohort of *C. marshallae* was higher in 2008 as compared to 2009 and 2010 (Fig. 6), implying that recruitment success to the diapausing cohort may have been more successful in 2008.

As typical of a Type I overwintering strategist, *C*. *marshallae* reproductive activity was focused in the spring period and by June most of the population was composed of stage C5 copepodites, presumably in diapause (Fig. 6). However, it appears that a fraction of the population did not enter diapause but continued to spawn. For example, a fraction of the stage C5 cohort of 2008 molted into adult females in early June (Fig. 6). This continued reproductive activity did not result in large cohorts of juvenile stages over the summer (Fig. 6). A second, major, reproductive event was apparent in August 2008 and 2009, when the majority of C5 copepodites molted into adult females (Fig. 6). No sampling was conducted in August 2010, but a similar pattern was observed in October 2010, albeit the fraction of C5 molting into females was less than in August (Fig. 6). The population was again diapausing as stage C5 copepodites from November to January (Fig. 6). Molting from stage C5 to C6 started in February in all years (Fig. 6).

*Acartia longiremis*: A phenological shift in the timing of the G_1_ adult female population was also apparent for *A. longiremis*. Female abundance peaked in late May in 2008 and 2010 and in early June in 2009 (Fig. 6, upper right panels). Maximum numbers of C1 copepodites occurred late April in 2008 and in late May in 2009 (Fig. 6). Assuming a development time of 26 days from stage C1 to C6 (Table 3), it is probable that these C1 cohorts developed into the observed G_1_ adult populations (Fig. 7, upper right panels). The sampling resolution in 2010 was too coarse to resolve a peak in juvenile stages, but, using the observed G_1_ adult female abundances, the estimated date of appearance of the C1 cohort was assessed as late April (Fig. 7). Abundance of *A. longiremis* G_1_ adult females were comparable between years (Fig. 6).

*Acartia longiremis*, as a Type II overwintering strategy copepod, displayed a long reproductive season. Stage C1 and C2 were present since the start of the sampling season in March of all years (Fig. 6), and data from DFO2 showed that reproduction was prominent at least until September in 2009 and until November in 2010 (Fig. 6). In January and February, the population consisted only of females (Fig. 6). Surprisingly, no juvenile stages were observed over the summer of 2008 (Fig. 6). This may have resulted from the fact that DFO2 is a single station and that as such it is affected by patchiness noise and between year changes in the inlet circulation.

### Modeled egg production rates

**Figure 8:**
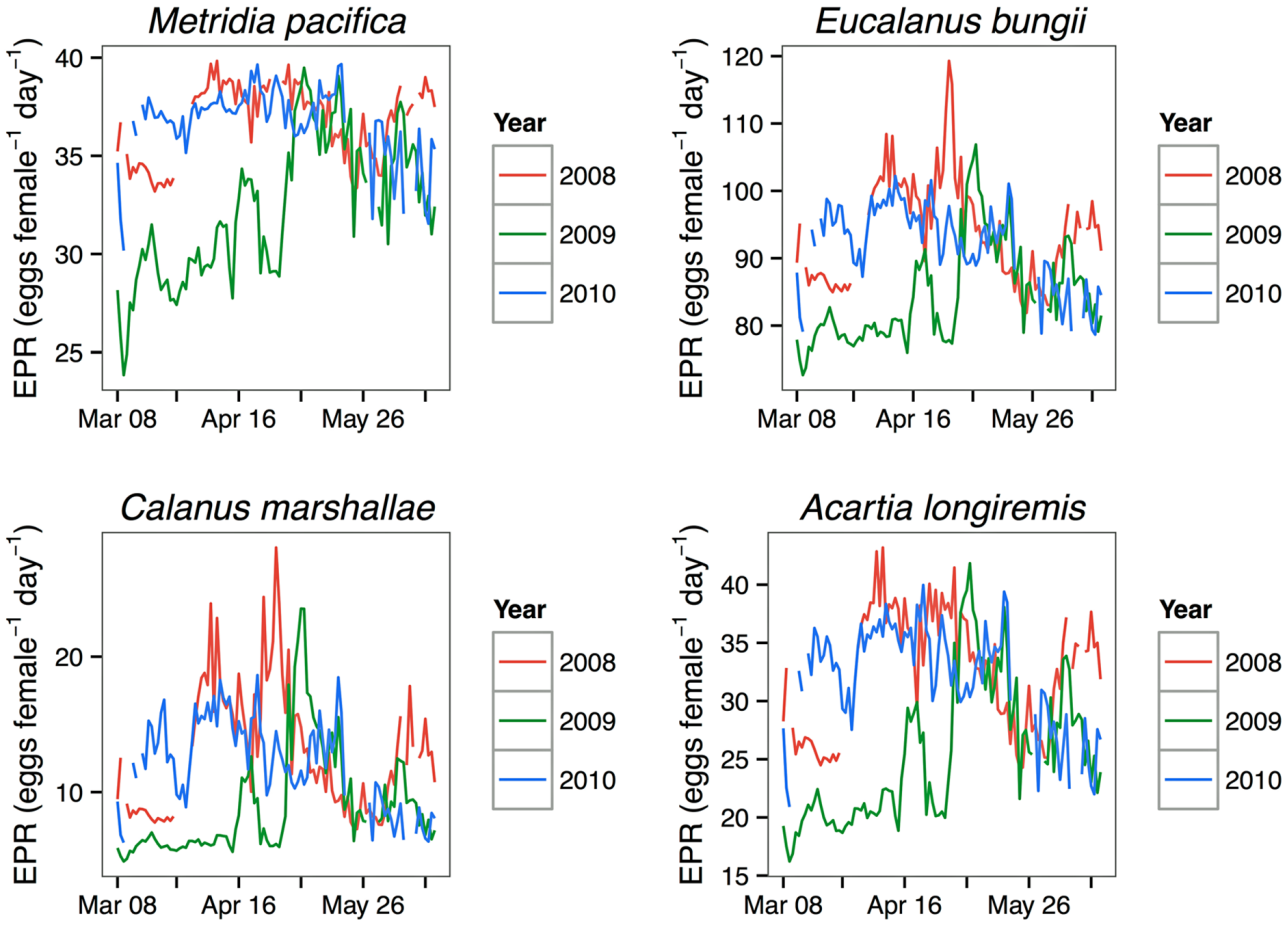
Seasonal variation in species-specific egg production rate estimates (egg female^−1^ day^−1^) at the Florence daily station.

**Figure 9:**
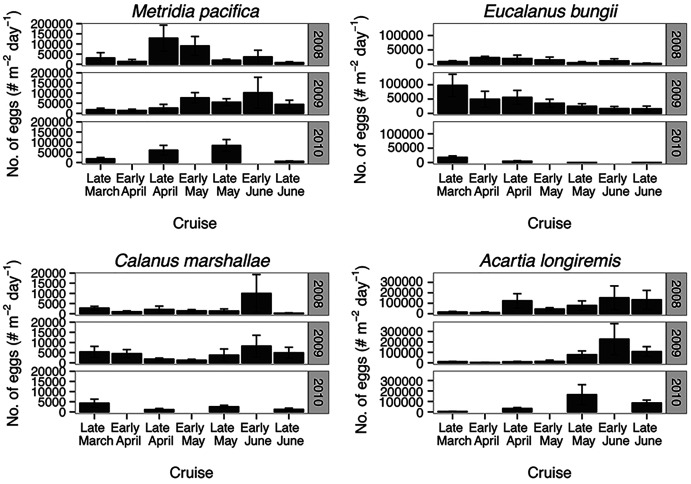
Average of egg abundance estimates (#m^−2^day^−1^) across inlet stations. Bars indicate standard error.

For all the copepod species under study, sustained maximum egg production rate (EPR) was observed from early April to late May in 2008 and 2010 and from early to late May in 2009 (Fig. 8). By contrast, the number of egg produced was dependent on the number of adult females present and varied between species. The number of eggs of *M. pacifica* started to increase from the low early spring values in late April in 2008 and 2010 and in early May in 2009 (Fig. 9). These spawning events likely produced the second cohort of stage C1 copepodites observed in late May of all years (Fig. 8). However, the birthdate of the G1 cohort was over the winter, a full 3–3.5 months before the bloom (Fig. 7). Thus, the *M*. *pacifica* G_1_ cohort did not result from income breeding of concurrent chlorophyll resources, but from capital breeding using stored lipids or income breeding with a feeding source other than chlorophyll.

*Eucalanus bungii* appeared to be dependent on the onset of the spring bloom for spawning initiation. Indeed, the late April peak in stage C1 *E. bungii* copepodites of 2008 and 2010 was a result of a spawning event in late March (Fig. 7), at the onset of the spring bloom (Fig. 3) and of increased EPR rates (Fig. 8), and high egg production (Fig. 9). By contrast, in 2009, while the estimated number of eggs produced was highest in late March (Fig. 9) as there were more females at the surface at the start of the season (Fig. 6), the estimated birthdate of the observed G_1_ C1 cohort occurred in early May at the onset of the 2009 spring bloom (Fig. 7) and of high EPR (Fig. 8). The lack of C1 copepodites prior to early May 2009 further indicates that *E. bungii* may rely on the onset of the spring bloom for spawning.

By contrast, *C. marshallae* appeared to start reproduction prior to the bloom, but copepodites were recruited to the diapausing cohort only after the bloom. The observed early April stage C1 cohort in 2009 developed from eggs spawned in late March 2009 (Fig. 9). Similarly, the observed late March stage C1 copepodites of 2008 and 2010 would have resulted from an early spawning event in early March. However, these pre-bloom C1 cohorts did not successfully develop into C5 (Fig. 6). The observed G_1_ C5 cohort developed instead from eggs produced in April in 2008 and 2010 and in early May in 2009 (Fig. 7) at the onset of the bloom when EPR was highest (Fig. 8).

*Acartia longiremis* behaved as an income breeder, with the entire G_1_ population developing from eggs spawned during the bloom. When the bloom was a month later (Fig. 3), the appearance of the G_1_ cohort occurred a month later (Fig. 7). Given the 22-day development time from egg to stage C1 and our 15 days sampling frequency, the peak in C1 copepodites observed in late April 2008 and late May 2009 (Fig. 6) resulted from the increase in egg production rate in early April 2008 and early May 2009 (Fig. 8).

## Discussion

### Relationship between phenology, overwintering strategy, and spring environmental forcing

Phenology of all the copepod species under study was delayed when the spring bloom occurred later. Thus, even the seasonality of omnivorous and carnivorous Type II copepods must be indirectly linked to the seasonality in phytoplankton production. Indeed, even when the timing of seasonal succession in zooplankton community composition was delayed in years characterized by a later phytoplankton bloom, the same zooplankton community composition was observed in Rivers Inlet over the period 2008–2010 (Tommasi et al., 2013).

For *Paraeuchaeta elongata*, the phenology shifts only applied to its early life stages, with adult densities remaining constant over the spring period. Alonzo, Mayzaud & Razouls (2000) and Auel & Hagen (2005) observed that *P*. *antarctica* reproduction increased in spring, possibly as a result of improved feeding conditions following the ontogenic ascent and reproduction of overwintering copepods. In the Skagerrak, *P*. *norvegica* consumes 6–14% of copepod population spring production (Tonnesson, Nielsen & Tiselius, 2006). The observed increase of *P*. *elongata* C1 copepodites in early spring in Rivers Inlet is consistent with enhanced feeding conditions in late winter and early spring, when *M. pacifica* and later *C*. *marshallae* exited diapause and commenced reproduction.

*Metridia pacifica* displayed the earliest cohort timing of any species. The appearance of the first annual cohort of stage C1 copepodites always occurred approximately 45 days prior to the bloom and its birthdate was consistently 3 months before the bloom. For a continuously reproducing copepod species, appearance of a distinct cohort may be a result of an increase in bottom-up driven rates, such as egg production, or of a decrease in top-down processes, such as predation mortality. We showed that, in all years, the second cohort of *M. pacifica* may have been an example of an increase in egg production rate following an increase in chlorophyll. However, the *M. pacifica* G_1_ cohort was spawned in winter, when egg production was at its lowest, suggesting that survival may have controlled the appearance of this cohort. Cohort development of *M. lucens* in the North Atlantic was negatively related with *C. finmarchicus* abundance through intraguild predation of *C. finmarchicus* on the early stages of *M. lucens* (Irigoien & Harris, 2006). The birthdate of the *M. pacifica* G_1_ cohort occurred prior to the ontogenic migration of *C. marshallae* in every year, suggesting that a similar mechanism may have been at play in Rivers Inlet. Unlike *P. elongata*, *M. pacifica* is a surface spawner (Padmavati, Ikeda & Yamaguchi, 2004) and thus its eggs may be vulnerable to predation from newly molted *C. marshallae* females. A careful assessment of *M*. *pacifica* winter and early spring mortality rates, predation pressure, and feeding history in relation to the spring phytoplankton bloom should be undertaken to determine the potential mechanism of the observed *M*. *pacifica* phenological variation.

A later onset of the spring bloom was associated with a delay in *A. longiremis* peak egg production rates, and resulted in a phenological shift in the appearance of its G_1_ cohort. Adult *A*. *longiremis* abundance peaked at the end of the spring bloom, when dinoflagellates dominated the phytoplankton community composition (Margalef, 1978). A later *Acartia* spp. biomass peak following a later dinoflagellate phenology was also observed in the North Sea (see review by Mackas et al., 2012). Since the observed differences in development times resulting from the interannual variation in spring temperature cannot account for the 1 month interannual difference in the timing of appearance of peak stage C1 densities and since phenology of *A. longiremis* was comparable in 2008 and 2010, 2 years of differing spring temperatures, we conclude that in Rivers Inlet an earlier buildup of the population is more dependent on an earlier timing of the spring bloom, and the associated high egg production rates, than on temperature. However, *Acartia* spp. phenology in other regions of the North Atlantic and in the Mediterranean appears to be more strongly associated with SST than phytoplankton phenology (see review by Mackas et al., 2012). Such regional differences may arise from variation in the overwintering strategy of the dominant *Acartia* species. Most *Acartia* species overwinter as resting eggs, whose hatching is temperature dependent (Miller, 2004). By contrast, *A. longiremis* diapauses in low numbers as immature females (Norrbin, 2001).

Similarly to *A. longiremis*, the dependence of *E. bungii* on spring bloom initiation for spawning resulted in a phenology shift in the timing of G_1_ copepodites following a shift in spring bloom timing. However, while in 2008 and 2010 the egg production pattern coincided with the timing of high egg production and cohort timing, in 2009 it did not. The estimated egg numbers were actually highest in late March 2009 because of high female abundance. The discrepancy between egg production estimates and the timing of appearance of the C1 cohort, and the lack of C1 copepodites until early May 2009, suggests that egg mortality was extremely high or that females were immature pre-bloom. In the Oyashio region significant numbers of mature *E. bungii* females only appeared at the onset of the spring bloom (Shoden, Ikeda & Yamaguchi, 2005; Yamaguchi et al., 2010), suggesting that *E. bungii* females may indeed have been immature prior to the bloom.

A shift in *E. bungii* cohort timing may also have resulted from a later emergence from diapause. Indeed, a larger fraction of the population comprised adult females in February 2008 as compared to 2009, suggesting that moulting from C5 to adults occurred earlier in 2008 than 2009. The cue to exit diapause and initiate moulting in *E*. *bungii* is unknown. However, temperature has been proposed as an environmental control of diapause emergence and maturation in other copepod species (Mackas et al., 2012). Thus, the later maturation in 2009 may be related to the lower temperatures of the deep layers in late winter 2009. By late March water temperatures below 50 m were comparable between years, and most of the population had molted into adult females also in 2009. Nevertheless, no stage C1 copepodites were observed until early June 2009, thus we suggest that a temperature-driven shift in the timing of emergence was not a likely driver of the observed changes in cohort timing.

Phenology of *C. marshallae* appears to be driven by differences in survival rather than changes in egg production. Reproduction started before the bloom, but recruitment was successful only for the cohort that developed into early copepodites during the spring bloom. These results are in agreement with observations from the Norwegian Sea demonstrating that *C*. *finmarchicus* copepodites do not develop beyond the early copepodites stages until the spring bloom (Hirche, Brey & Niehoff, 2001), and with observations from the Bering Sea indicating that fitness is highest for those *C*. *marshallae* C1 copepodites appearing at the onset of the bloom (Baier & Napp, 2003). Thus, in Rivers Inlet, like in the Bering Sea, *C*. *marshallae* phenology appears to be controlled by differential survival of early copepodites rather than production and is dependent on the timing of the spring bloom.

Similarly to *E. bungii*, a delay in phenology of C. *marshallae* may also have been driven by interannual changes in temperature. However, the timing of the terminal molt of *C*. *marshallae* was not later when water temperatures were colder in 2009; the fraction of adult females relative to stage C5 copepodites was actually higher in February 2009 as compared to February 2008. Therefore, internal controls of timing of diapause emergence such as accumulated lipid reserves may be more important than direct temperature control in determining the timing of emergence of *C*. *marshallae* C5 copepodites, as was concluded by Johnson et al. (2008) for *C*. *finmarchicus*. This suggests that temperature was not a direct driver the observed variation in *C. marshallae* phenology. Nevertheless, these results are in disagreement with data from the North Sea (see review by Mackas et al., 2012), showing that sea surface temperature may be a more important forcing of C. *finmachicus* phenology than phytoplankton bloom timing. This may be due to site-specific differences in the seasonality of phytoplankton dynamics, the seasonality of predation pressure, or the relative importance of advection as a driver of population dynamics. In Rivers Inlet, advection was not the main driver of *C*. *marshallae* population dynamics as compared to local demographic processes (Tommasi et al., 2014).

### Relationship between recruitment success, overwintering strategy and spring environmental forcing

In terms of recruitment success, the response of the copepods under study was specific to each type of overwintering strategy. While the phenology of all copepod species was delayed following a later bloom, a delay in bloom timing was only associated with poorer recruitment success of Type I copepods. Reduction in recruitment of *E. bungii* and *C. marshallae* following a delay in spring bloom timing has also been observed elsewhere (Baier & Napp, 2003; Kobari et al., 2007). Furthermore, only Type I copepods displayed declines in G_1_ cohort size at high temperatures, during the El Niño year. It appears that continuous reproduction and an omnivorous feeding strategy allows Type II copepods to exploit seasonally variable food resources and to build their populations to a comparable size during years of varying spring environmental forcing and spring bloom timing. By contrast, species that focus their reproductive effort over the spring period appear more vulnerable to interannual variation in spring forcing.

Differential response of copepods to environmental forcing has long been recognized in the Northern California Current system, where Northern copepods dominate over Southern types in relation to ocean transport (Hooff & Peterson, 2006; Mackas, Batten & Trudel, 2007; Keister et al., 2011). However, most of the species considered here belong to the Northern group. More specifically, *E. bungii* and *M. pacifica* are subarctic oceanic copepods and *C. marshallae* and *A. longiremis* are boreal shelf copepods (Mackas, Batten & Trudel, 2007). *P. elongata* has generally not been associated with either Northern or Southern copepods. Our results are the first to demonstrate that further changes in community composition may be superimposed over the observed transport-driven variation following shifts in environmental forcing. This pattern may be particularly important for northern regions, which are presumably sources of these copepods, as well as for coastal embayments, such as fjords or inland seas, whose zooplankton community may not be as impacted by the circulation over the shelf break.

While it was not an aim of this study to assess the mechanisms for the observed variation in recruitment, some generalizations on the potential drivers of the observed pattern can be made. The 2009 spring bloom was less prolonged. Earlier termination of the bloom may have reduced growth rates of both *E. bungii* and *C. marshallae* copepodites (Campbell et al., 2001; Takahashi & Ide, 2011), and have resulted in a smaller diapausing cohort. However, the 2010 bloom was similar in duration to 2008, suggesting that top-down processes, rather than bottom-up processes played a role in determining the size of the diapausing C5 cohort. Recent studies have shown that mortality may be an important driver of copepod recruitment (Li et al., 2006; Plourde, Maps & Joly, 2009; Ji, Stegert & Davi, 2013). Thus, overall recruitment of both *E. bungii* and *C. marshallae* may have been lower when the bloom was later because of higher cumulative mortality of adult females from the time of diapause emergence to the time of successful copepodite recruitment during the bloom. Higher mortality of copepodites with a later birthdate, when temperature was higher, may also explain the lower recruitment when the bloom was late. The substantial decrease in Type I recruitment during the El Niño year provide further support for the hypothesis of higher mortality of Type I copepods at higher temperatures. The number of invertebrate predators increases over the season (Tommasi et al., 2013), predation rate increases with temperature (Davis, 1984; Ji et al., 2009), suggesting that mortality of Type I copepods may have been higher at higher temperatures. Indeed, the short reproductive period of Type I copepods may well have evolved as a response to higher predatory rates over the summer period. Since summer plankton appears to respond more strongly to changes in sea surface temperature than copepods dominating the spring community (Edwards & Richardson, 2004), an assessment of the potential for an increase in the overlap of high predation and Type I copepod at high spring temperatures is warranted. Future research should also assess seasonal and interannual variation in predator densities in relation to temperature and phytoplankton dynamics to assess the potential for top-down control of *E. bungii* and *C. marshallae*.

Both *E. bungii* and *C. marshallae* had a second major spawning event in late summer/early fall. It remains to be assessed how phenology of fall spawning events changes interannually and how important fall spawning events are to recruitment in the following spring. If the successful recruitment window of Type I copepods in spring, marked by high chlorophyll concentrations and low mortality, will be reduced under future climate change scenarios, fall recruitment events may become essential to the maintenance of the annual reproductive success of these species.

Since recruitment success of fish larvae relies on synchronization with peaks of their zooplankton prey (Cushing, 1969; Hjort, 1914), the observed phenological variation in zooplankton dynamics may result in phenologically driven ecosystem shifts. Recruitment success of upper trophic levels may be further impaired in years of a delayed spring bloom or high spring temperatures by declines in Type I copepods, an energy rich prey (Mackas, Batten & Trudel, 2007; Trudel et al., 2007). Future studies should evaluate direction of climate driven changes in spring forcing events and their effect on both zooplankton phenology and that of upper trophic levels to assess the potential for a significant mismatch between upper trophic levels and their zooplankton prey in the future.

## Supplemental Information

10.7717/peerj.12238/supp-1 Supplemental Information 1 Sampling periods in Rivers Inlet.

10.7717/peerj.12238/supp-2 Supplemental Information 2 Logarithmic regression of the EPR and chlorophyll concentrations reported in Smith & Lane (1991) for *Eucalanus californicus* used to model the relationship between *Eucalanus bungii* EPR and chlorophyll. The equation is presented in Table 2.

10.7717/peerj.12238/supp-3 Supplemental Information 3 Species-specific average egg production rate (eggs female^−1^ day^−1^) across inlet stations in 2010 computed for each zooplankton depth sampling interval. Error bars represent standard errors. AL = *Acartia longiremis*, CM = *Calanus marshallae*, EB = *Eucalanus bungii*, MP = *Metridia pacifica*.

## Additional Information and Declarations

### Data Availability

The following information was supplied regarding data availability: The raw data for the analysis are parameters reported in Tables 1 and 2.
