# Supplementary material for: Differential response of distinct copepod life history types to spring environmental forcing in Rivers Inlet, British Columbia, Canada
Source: PeerJ. 2021 Oct 18;9:e12238. doi: 10.7717/peerj.12238 (PMC8530099; doi:10.7717/peerj.12238)
Supplement: Supplemental Information 1 [file peerj-09-12238-s001.pdf]

Supplementary Table 1: Sampling events in Rivers Inlet during 2008, 2009 and 2010 surveys. Only bolded and highlighted (most consistent sampling) period is used in the paper.

**Time interval**	**2008**	**2009**	**2010**
Late February	NA	February 26-27	NA
**Late March**	**March 18-19**	**March 17-18**	**March 18-19**
**Early April**	**March 31-April 1**	**April 3-4**	**NA**
**Late April**	**April 23-24**	**April 16-17**	**April 23-24**
**Early May**	**May 9-10**	**May 4-5**	**NA**
**Late May**	**May 25-26**	**May 19-20**	**May 19-20**
**Early June**	**June 8-9**	**June 2-3**	**NA**
**Late June**	**June 24-25**	**June 17-18**	**June 21-22**
Early July	July 9-10	July 2-3	NA
Late July	July 22-23	July 17-18	NA
August	August 4-5	August 14-15	NA
September	September 23-24	September 16-17	September 12-13
