# Supplementary figures and images for: Differential response of distinct copepod life history types to spring environmental forcing in Rivers Inlet, British Columbia, Canada
Source: PeerJ. 2021 Oct 18;9:e12238. doi: 10.7717/peerj.12238 (PMC8530099; doi:10.7717/peerj.12238)

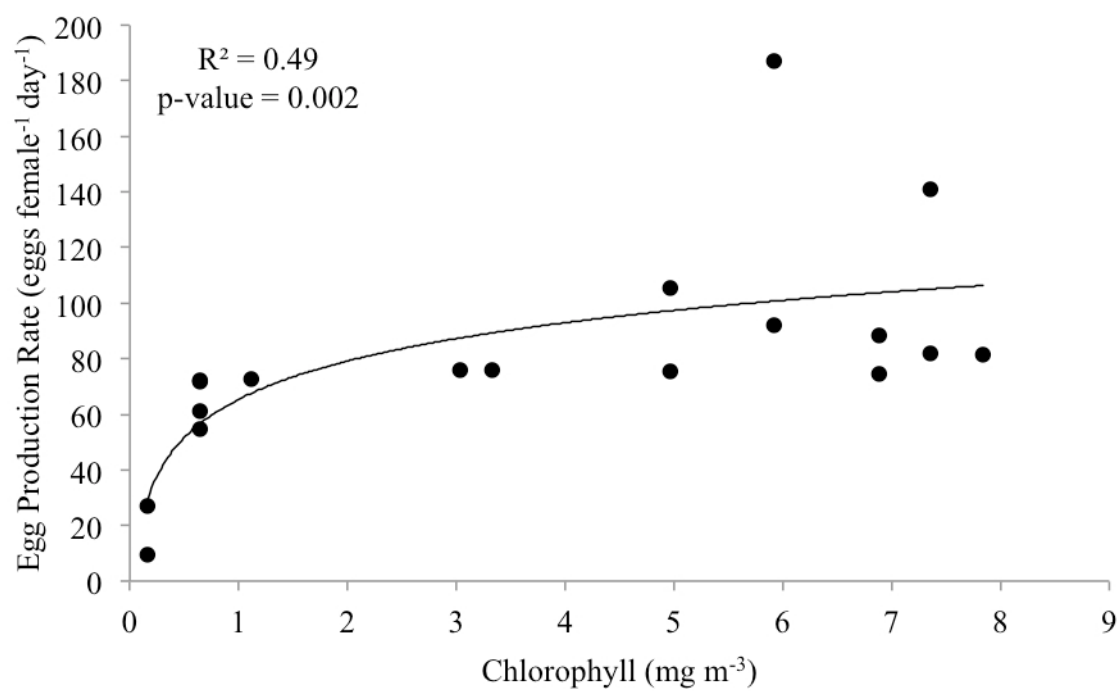

Appendix 1.

Supplement: Supplemental Information 2 — The equation is presented in Table 2. [file peerj-09-12238-s002.pdf]

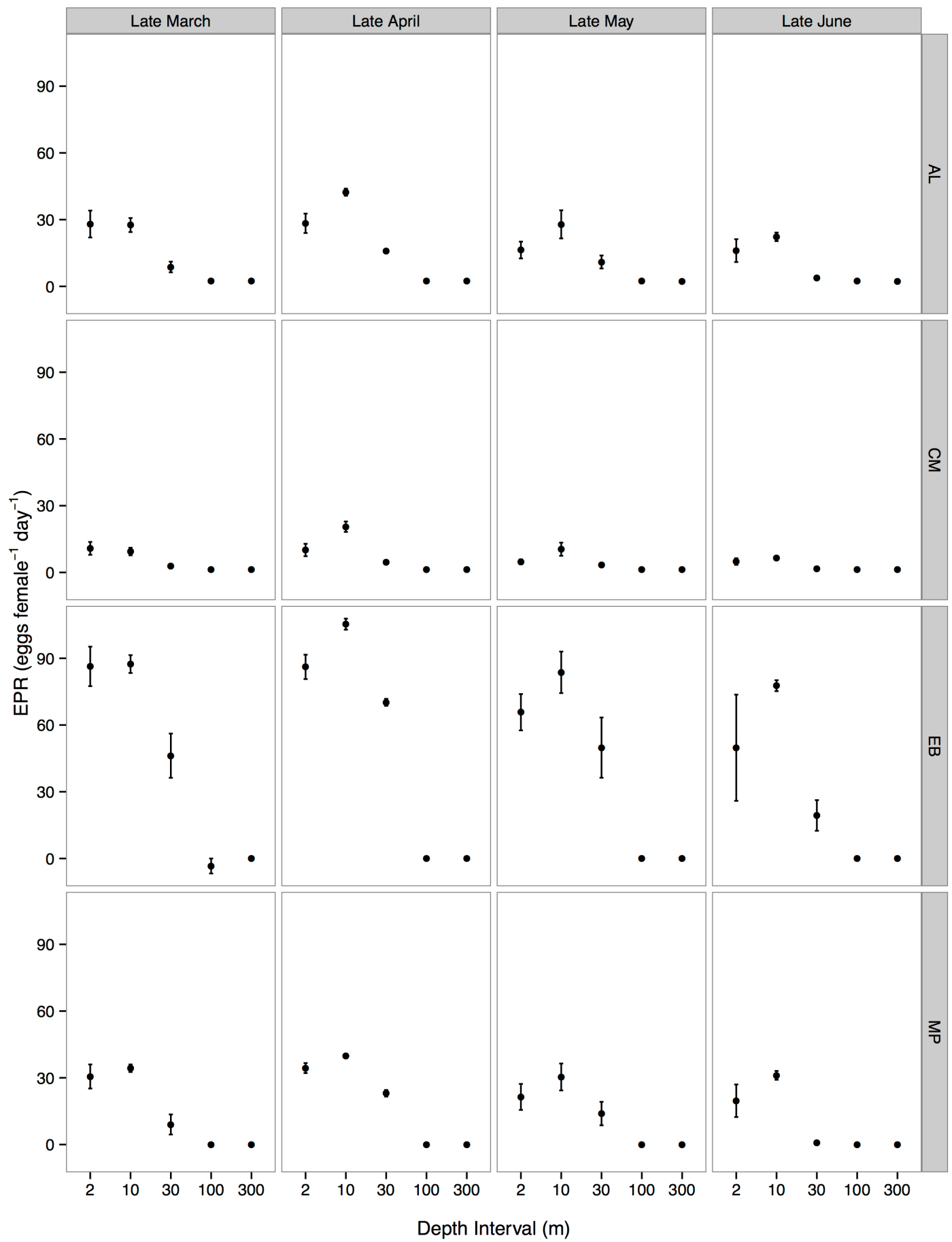

Supplement: Supplemental Information 3 — Error bars represent standard errors. AL = Acartia longiremis, CM = Calanus marshallae, EB = Eucalanus bungii, MP = Metridia pacifica. [file peerj-09-12238-s003.pdf]
